# Effects of elevated emotional symptoms on metabolic disease development: a 10-year follow-up study

**DOI:** 10.3389/fpsyt.2023.1148643

**Published:** 2023-12-04

**Authors:** Yolanda Sanchez-Carro, Alejandro de la Torre-Luque, Christina Vassou, Pilar Lopez-Garcia, Ekavi Georgousopoulou, Christos Pitsavos, José Luis Ayuso-Mateos, Demóstenes Panagiotakos

**Affiliations:** ^1^Department of Psychiatry, Universidad Autonoma de Madrid, Madrid, Spain; ^2^Department of Psychiatry, Instituto de Investigación Sanitaria Princesa (IIS Princesa), Madrid, Spain; ^3^Center for Biomedical Research in Mental Health (CIBERSAM), Carlos III Health Institute, Madrid, Spain; ^4^Department of Legal Medicine, Psychiatry and Pathology, Universidad Complutense de Madrid, Madrid, Spain; ^5^School of Health Sciences and Education, Harokopio University, Athens, Greece; ^6^First Cardiology Clinic, School of Medicine, University of Athens, Athens, Greece

**Keywords:** depression, anxiety, inflammation, metabolic syndrome, diabetes, hypertension, hypercholesterolemia

## Abstract

**Background:**

In recent decades, the relationship between emotional disorders (i.e., depression and anxiety) and alterations in physiological functions (i.e., inflammation or metabolism) have been well supported. However, studies on a symptom-based approach have provided mixed results. Our study aims to gain insight into how subclinical statuses, featured by elevated depressive and/or anxious symptoms, may influence immunometabolic alterations in the concurrent relationship; and the development of metabolic diseases at 10-year follow-up: diabetes, hypertension and hypercholesterolemia.

**Methods:**

Data from 758 Greek adults [394 men (aged 41 ± 10 years) and 364 women (aged 37 ± 12 years)] were used. Four groups were created according to the levels of depressive and anxiety symptoms: (1) control group (CG), (2) depressive group (DG), (3) anxiety group (AG) and (4) depressive and anxiety group (DAG). Multi-indicator multi-causes (MIMIC) modeling was used to estimate metabolic function and inflammatory response scores, on a wide selection of blood biomarkers. Finally, a binary logistic regression was carried out to study the influence of symptoms on the development of the aforementioned metabolic diseases on a 10-year follow-up.

**Results:**

Group membership was not associated with metabolic function score. Conversely, DAG membership was related with higher inflammatory response score (*B* = 0.20, *CI*_95_ = 0.01, 0.40), with respect to the CG (*p* < 0.05). Both age and sex were significant variables in the calculation of both scores. Regarding disease at 10-year follow-up effect, risk of developing diabetes, hypertension and hypercholesterolemia was associated with age and socioeconomic status. Moreover, DG membership was significant for diabetes risk (*OR* = 2.08, *CI*_95_ = 1.00, 4.22) and DAG for hypercholesterolemia (*OR* = 1.68, *CI*_95_ = 1.16, 2.43).

**Limitations:**

Data on anti-inflammatory drugs and psychopharmacological medication were not collected in this study.

**Conclusions:**

Elevated symptoms of depression and anxiety accounts for inflammatory alterations at concurrent relationship and a higher risk of 10-year follow-up metabolic diseases.

## 1 Introduction

Emotional disorders (i.e., depression and anxiety) are considered among the top five mental conditions with higher impact, worldwide. In 2015, it is estimated that 4.4% of the world population suffered from a depressive disorder and 3.6% from an anxiety disorder (1). Moreover, comorbidity is quite common between both conditions, observing shared pathophysiological mechanisms and risk factors (2–4). Importantly, emotional disorders may account for large proportion of years of life lived with disability across the lifespan (5, 6).

Emotional disorders may have a critical impact for patients at the individual level, as well as for healthcare provision systems at the socioeconomic level. Further research should be done to improve the understanding of pathophysiological mechanisms to contribute for treatment optimization. In last decades, mounting evidence has stressed the existing relationship between depression and inflammation (7–10). In this line, some depressive endophenotypes (i.e., atypical depression) have been related to evident disturbances in the (pro-)inflammatory response (11–13). Less is known on the relationship between anxiety and inflammation (14). However, existing studies also point to an elevated pro-inflammatory response in patients with an anxiety disorder (10, 15). This inflammation could lead to the occurrence of metabolic diseases comorbid with depression and anxiety, as it seems to have a mediating role in both pathologies, for example, patients with depression are at higher risk of high blood pressure and patients with type 2 were 1.2–2.3 times more likely to have depressive symptoms than the general population (16). The relationship between the two (i.e., emotional disorders and metabolic diseases) seems to be mediated by inflammation. For instance, some evidence stressed a mediating role of NLRP3 inflammatory bodies in hippocampal neuroinflammation and depression-like behavior (16, 17). Thus, the metabolic alteration, would be preceded by an altered inflammatory status.

Unfortunately, studies on comorbid anxiety and depression are scarce. It would be expected to find wider inflammatory alterations in patients with comorbid emotional disorders for several reasons. First, patients with inflammatory diseases have an increased risk of developing both anxiety and depressive disorders (18, 19). Second, both emotional disorders may share some common altered mechanisms. A dysregulation of the hypothalamic pituitary adrenal axis (HPA) is observed in both disorders (20–22), which becomes more evident when they occur together (23).

Dysregulation of the HPA axis, seems to play a role in the immunometabolic alterations observed in patients, such as increased production of cytokines [i.e., Tumor necrosis factor (TNF-α) or interleukin 6 (IL- 6)] (24) and consequent induction of acute phase inflammatory proteins [i.e., and C-reactive protein (CRP) or serum amyloid A (SAA)] (25) and metabolic dysregulation [i.e., stimulation of the release of lipids into the bloodstream, increased triglycerides and decreased cholesterol linked to high-density lipoproteins (HDL-C) or alterations in total cholesterol levels] (26). Moreover, the dysregulation of cortisol secretion may induce alterations in lipid and glucose metabolism that can contribute to metabolic disease development, such as diabetes, hypercholesterolemia or hypertriglyceridemia (27). Evidence is less consistent on the development of cardiovascular conditions and it seems that other mediating pathways (i.e., kynurenine path) may be involved (28).

Although most of the available studies are cross-sectional (8, 9, 14), the longitudinal studies carried out to date seem to relate the presence of emotional disorders with immunometabolic alterations in the short and long term (23, 29–35). Moreover, short-term alterations in the immunometabolic response have also been observed among individuals with subclinical emotional conditions (36–39). Unfortunately, longitudinal studies are scarce on the relationship between subclinical (symptom-based) emotional conditions and metabolic disease development.

The study of subclinical conditions become crucial for prevention to tackle the development of full-blown conditions and mitigate its impact over time. This study aimed to analyze the relationship between subclinical conditions featured by elevated emotional symptoms (i.e., depressive and/or anxious symptoms) and immunometabolic dysregulation at concurrent relationship (diverted levels of immunometabolic markers in plasma) and disease at 10-year follow-up. We hypothesize that participants with elevated levels of both depressive and anxious symptoms would show altered immunometabolic profiles in comparison with people without elevated symptoms. Moreover, it is hypothesized that the presence of comorbid depression-anxiety symptoms would contribute to the development of chronic metabolic diseases (particularly diabetes and hypercholesterolemia) over a 10-year follow-up.

## 2 Materials and methods

### 2.1 Study sample

Data from the 10-year follow-up of the ATTICA study (40) were used to satisfy the study aims. The ATTICA study is a population-based cohort focused on examining social, demographic, lifestyle, clinical and biological characteristics of apparently healthy Greek adults living in the greater metropolitan area of Athens (Greece), on cardiovascular disease incidence, and other health-related conditions. In brief, a baseline survey was carried out during 2001–2002 and a sample of 4056 adults was invited to participate (78% from urban area). This survey relied on a random, multi-stage cluster sampling (considering age and sex distribution of the Attica region in 2001). A total of 3042 individuals (18–89 years old, 49% men) agreed to participate (75% response rate). A follow-up survey was conducted during 2011–2012. Most of participants were enrolled the follow-up survey (*n* = 2583; 85% response rate).

A randomly selected subsample of the ATTICA cohort completed questionnaires on emotional symptoms. Concretely, a sample of 758 adults was used [394 men (aged 41 ± 10 years) and 364 women (aged 37 ± 12 years)]. Further details on how the subsample was reached and randomization algorithm are provided elsewhere (41). The final sample comprised 615 participants (50.98% women; *m* = 39.20 years, *sd* = 10.96). Participants were dropped out because: being older than 65 years at baseline (*n* = 3), blood sample not available (*n* = 137) or death at follow-up (*n* = 3). Significant differences were found between the random subsample initially selected and final sample in analysis, only in terms of family composition at baseline [χ^2^ (2) = 8.99, *p* < 0.05, Cramer’s *V* = 0.10], with higher proportion of married individuals in the final sample (62.74%) than the one from the drop-out sample (51.75%); and in terms of depressive symptom levels [*t* (204.66) = 2.09, *p* < 0.05, Cohen’s *d* = 0.2], with lower symptoms in the subsample in analysis (*m* = 35.12, *sd* = 7.35) in comparison to the drop-out one (*m* = 36.62, *sd* = 7.82).

### 2.2 Demographic, clinical and lifestyle characteristics

Baseline survey included questions about demographic features (i.e., gender, age, marital status, education level and financial status), anthropometric measures [i.e., height, weight, and body mass index (BMI)], history of medical conditions as well as lifestyle habits (i.e., dietary assessment, alcohol consumption, tobacco use and physical activity). The assessment protocol has already been described elsewhere (42).

Depressive symptoms were assessed using the validated Greek translation of the Zung’s Self-Rating Depression Scale (ZDRS) (43, 44). ZDRS consists of 20 items covering affective, psychological, and somatic symptoms. To perform the assessment, the patient indicates how often they experience a particular symptom (i.e., 1 = some of the time, 2 = some of the time, 3 = a good part of the time, or 4 = most of the time). The ZDRS total score range is 20–80; with higher values indicating more severe depression symptoms.

Anxiety symptoms were assessed using the validated Greek translation of the Spielberger State Anxiety Inventory (STAI), which is a 20-item self-administered questionnaire (45). The 20 items are scored from 1 to 4 in terms of frequency categories with respect to (never, sometimes, many times, always) and the total scores are obtained by adding the values assigned to each response (46). The total score of the 20-item STAI ranges from 20 to 80 with higher score values being indicative of more severe anxiety symptoms (47).

### 2.3 Biochemical measurements

All participants were summoned on after 12 h of fasting, to carry out the blood test from 8 to 10 a.m. All the blood samples were collected under the same procedure (in a sitting position and were collected from the antecubital vein) and were carried out in the same laboratory that followed the criteria of the World Health Organization Lipid Reference Laboratories.

#### 2.3.1 Metabolic measures

The metabolic indicators were selected according to the National Cholesterol Education Program’s Adult Treatment Panel III (revised) report guidelines for metabolic syndrome (MetS) (48). Triglycerides, high density lipoprotein (HDL)-C and blood sugar levels were quantified to know the metabolic state of participants. These biochemical examinations were measured using chromatographic enzymic method in a Technico Automatic Analyzer RA-1000 (Dade Behring Marburg, Germany). In addition, waist circumference, as well as systolic and diastolic blood pressure were measured to determine the metabolic risk factors.

For assessing the validity of the methods details may found elsewhere (41).

#### 2.3.2 Inflammatory measures

The blood samples for the inflammatory biomarkers were taken under the same procedure, at the same time and place as the samples collected for the analysis of metabolic markers. C-Reactive Protein (CRP) and serum amyloid – A (SAA) were assayed by particle-enhanced immunonephelometry (N Latex, Dade-Behring Marburg GmbH, Marburg, Germany) (49). Interleukin-6 (IL-6) levels were quantified with high sensitivity enzyme linked immunoassay (R&D System Europe Ltd., Abingdon, UK) and tumor necrosis factor (TNF)-α was measured with ELISA method (Quantikine HS/human TNF-α immunoassay kit, R&D Systems, Inc., Minneapolis, USA) (49).

### 2.4 Follow-up examination

The ATTICA Study’s investigators performed the 10-year follow-up (median follow-up time 8.41) [see in Georgousopoulou et al. (40)]. During follow-up, the presence or absence of diabetes, hypercholesterolemia and hypertension was determined as follows.

Regarding metabolic diseases, some standard criteria were adopted. First, diabetes diagnosis was determined by fasting blood glucose levels greater than 125 mg/dL or the use of antidiabetic medication. Hypercholesterolemia was defined as total serum cholesterol levels greater than 200 mg/dL or the use of lipid-lowering agents. The presence of hypertension was determined by values greater than or equal to 140/90 mmHg or by being under hypertensive medication. Blood pressure was measured with the participant sitting and resting for at least 30 min, the specialist doctor performed three measurements on the right arm, in a 45° position and leaning on the table with the aneroid manometric sphygmomanometer (ELKA, Von Schlieben Co., Munich, Germany). The level of systolic blood pressure was determined by the first perception of sound and the diastolic was determined by phase V when the repetitive sounds disappear completely [for more information on how the samples were collected, see (41)].

### 2.5 Data analysis

Multi-indicator multi-causes (MIMIC) modeling was used to estimate both a metabolic risk score and an inflammatory response score from the biomarkers. MIMIC modeling constitutes a Structural Equation Modeling (SEM) extension to study nested relationships, simultaneously allowing for identifying underlying (latent) factors that are measured by multiple indicators and controlling for other confounding effects (50). Thus, the metabolic risk score was estimated by means of blood (triglycerides, HDL-C, blood sugar, all of them in loglinear scale), cardiovascular (arterial blood pressure) and anthropometric (waist diameter) indicators. Likewise, the inflammatory response score was estimated by using blood indicators (CRP, SAA, IL-6 and TNF-α). Score estimation was conducted controlling for relevant lifestyle covariates (Mediterranean diet adherence, physical activity, smoking and alcohol use). The diagonally weighted least squares (DWLS) methods were used for model estimation, as some binary (e.g., hypertension diagnosis, smoking, alcohol use) and categorical (physical exercise) were included in our analysis. Standard error estimation was based on bootstrapping methods with 1000 samples, that ensures reliable estimates are derived. Fit indexes used to assess goodness-of-fit of MIMIC models were the χ^2^ statistic, the root mean square error of approximation index (RMSEA), the comparative fit index (CFI), the Tucker-Lewis index (TLI), and the standardized root mean square residual (SRMR). According to Hu and Bentler (51), adequate model fit is indicated by values of RMSEA < 0.08, CFI ≥ 0.95, TLI ≥ 0.95, and SRMR < 0.08.

For the purposes of our study, sample was categorized into four groups regarding levels of depressive and anxiety symptoms: (1) control group (CG), featured by low levels of depressive and anxiety symptoms; (2) depressive group (DG), comprising individuals with depressive symptom levels overpassing the third quartile of distribution cut-off point and low levels of anxiety symptoms; (3) anxiety group (AG), whose members showed low depressive symptoms but anxiety symptoms overpassing the third quartile of distribution cut-off point; and (4) depressive and anxiety group (DAG), whose members showed elevated levels of both anxiety and depressive symptoms.

Relationship between the metabolic risk and inflammatory scores derived from the MIMIC models (i.e., predicted scores) and study group membership was studied using linear regression. Sex and age were used as covariates. The adjusted *R*^2^ was used as an effect size estimate. Beta coefficients and their CI_95_ was used to explore loading magnitude.

To predict the development of metabolic diseases (i.e., diabetes, hypertension and hypercholesterolemia) over the 10-year follow-up, logistic binary regression was used. Participants with suspected baseline diabetes (glucose level > 126 mg/dL; *n* = 15), hypertension (systolic blood pressure ≥ 140 or diastolic blood pressure ≥ 90; *n* = 172) or hypercholesterolaemia (total cholesterol level > 240; *n* = 57) were removed from analysis for the 10-year outcome. Taking into account the metabolic risk scores at baseline allows to control the levels of markers associated with metabolic alterations, as an additional adjustment following the exclusion of participants with suspected baseline disease for each outcome of interest.

Invariant (sex), and baseline covariates (age, financial status, emotional group and metabolic score) were considered. The baseline inflammatory response score was considered as a weighting factor for the within-subject heteroscedasticity structure, due to the potential influence on the development of both metabolic conditions and emotional disorders.

The Akaike information criterion (AIC) was used to compare the fit of an unconstrained model (model without covariates), a model with sociodemographic covariates (age and financial status) and a model with all the covariates (full model: age, financial status, emotional group and metabolic score). A better fit was proven by a lower AIC of the full model in comparison to the unconstrained one. In addition, the area under the receiver operating curve (AUC) was used as a classification accuracy estimate. AUC > 0.70 indicates adequate accurate in classification. The odds ratio (*OR)* estimate was used to explore loading magnitude.

All analyses were performed using the R software × 64 3.0.1 (l cmm, ROCR, psych and lmerTest packages).

## 3 Results

The descriptive statistics are displayed in Table 1, as well as group comparison according to their scores in depressive and anxious symptom scales: CG (61.32% of sample), DG (13.24), AG (11.52%) and DAG (13.90%) (see Table 1).

**TABLE 1:** Sociodemographic, clinical and lifestyle factors according to study group *(n* = 755).

	CG		DG		AG		DAG			
	(*n* = 463)		(*n* = 100)		(*n* = 87)		(*n* = 105)		Contrast test	ES
	*m*/%	*sd*	*m*/%	*sd*	*m*/%	*sd*	*m*/%	*sd*		
Sex (%male)	58.1		32		62.07		34.29		39.63*	0.23
Age (years)	39.54	10.26	36.31	12.25	41.83	10.89	38.31	12.06	0.11	0.01
Formal education (years)	13.51	3.27	13.29	2.92	12.18	3.57	11.77	3.54	29.17**	0.04
**Marital status**									17.04**	0.11
Never married	31.1		49		28.74		31.43			
Married	63.93		44		62.07		60.95			
Divorced/widowed	4.97		7		9.2		7.62			
**Household income**									32.78	0.12
1st quartile	16.04		28.28		25.58		34.95			
2nd quartile	26.15		32.32		22.09		28.16			
3rd quartile	36.26		30.3		33.72		24.27			
4th quartile	21.54		9.09		18.6		12.62			
Depression symptoms^1^	31.4	4.61	43.5	3.47	34.33	3.83	46.19	5.68	531.05**	0.41
Anxiety state^2^	34.9	7.96	39.43	6.95	55.13	4.81	58.16	6.56	1095.28**	0.59
Mediterranean diet adherence^3^	26.62	7.09	28.47	9.44	25.78	7.07	28.59	8.61	3.07	0
**Physical activity level**									14.4*	0.1
Low	31.1		49		28.74		31.43			
Moderate	67.82		51		70.11		66.67			
Intense	1.08		0		1.15		1.9			
Smoking (%yes)	60.69		56		60.92		56.19		1.33	0.04
Alcohol drinking (%yes)	91.79		82		88.51		85.71		10.03*	0.12
**Metabolic markers**										
Waist circumference (cm)	89.06	15.51	85.39	17.63	91.82	17.36	86.23	17.03	0.81	0
Fasting glucose (mg/dl)	87.87	16.54	89.05	27.4	89.69	15.7	89.45	29.68	0.84	0
Triglycerides (mg/dl)	102.43	63.01	96.36	55.6	111.42	67.41	100.08	70.88	0.01	0
HDL cholesterol (mg/dl)	47.91	12.52	52.96	13.73	46.62	15.17	49.39	13.57	0.64	0
SBP (mmHg)	118.01	16.13	114.99	15.13	119.17	15.54	115.1	19.68	1.43	0
DBP (mmHg)	78.69	11.41	75.65	11.63	80.12	11.92	75.91	11.41	2.39	0
**Inflammatory markers**										
CRP (mg/l)	1.74	2.21	1.73	2.57	2.32	2.9	2.28	3.03	6*	0.01
IL-6 (mg/dl)	0.31	0.2	0.27	0.25	0.35	0.2	0.31	0.25	0.84	0
SAA	3.63	4.42	4.09	3.16	3.64	4.22	3.56	2.61	0	0
TNF-α (mg/dl)	6.40	2.73	6.22	3.81	6.56	2.78	6.19	3.03	0.17	0
**Metabolic diseases at follow-up**									
Diabetes (%yes)	4.20		9.26		10.2		4.65		4.18	0.05
Hypercholesterol (%yes)	55.66		56.67		59.32		66.13		2.41	0.04
Hypertension (%yes)	44.37		32.26		52.46		42.86		5.27	0.06

Means (*m*) and standard deviations (*sd*) are displayed for continuous variables. Percentage (%) of cases is displayed for either dichotomous or categorical variables. The contrast test statistic was the *F* statistic for continuous measures and the χ^2^ statistic for either dichotomous or categorical variables. The effect size (ES) estimate was the η^2^_*partial*_ for continuous measures and the Cramer’s V statistic for either dichotomous or categorical variables. HDL, high-density lipoprotein; SBP, systolic blood pressure; DBP, diastolic blood pressure; CRP, C-reactive protein; iL-6, Interleukin 6; SAA, serum amyloid A; TNF-α, tumor necrosis factor α; CG, control group; DG, high depression group; AG, high anxiety group; DAG, high depression and anxiety group.

^1^Measured by the Zung Self-Rating Depression Scale.

^2^Measured by the State-Trait Anxiety Inventory, state form.

^3^Measured by the Mediterranean Diet Adherence Screener test.

*p < 0.05;

**p < 0.01.

Regarding the sociodemographic variables, we observed significant differences between the study groups. There was a higher percentage of men with anxiety (62.07%) compared to those in the DG and DAG groups, in which there was a higher percentage of women (68 and 65.71%, respectively). Significant differences were also observed in the variable of years of schooling (*F* = 29.17; *p* < 0.01; η^2^_*partial*_ = 0.04) and in marital status (χ^2^ = 17.04; *p* < 0.01; Cramer’s *V* = 0.11). The CG had more years of schooling (*m* = 13.51; *sd* = 3.27) than the rest of the participants, being participants from the DAG those with the lowest number of years of schooling (*m* = 11.77; *sd* = 3.54).

Regarding the emotional symptoms, between-group differences were evident for both the depression (*F* = 531.05; *p* < 0.01; η^2^_*partial*_ = 0.41) and the anxiety symptoms (*F* = 1095.28; *p* < 0.01; η^2^_*partial*_ = 0.59). In terms of lifestyle factors, there were significant differences, both in physical exercise and in current alcohol consumption. The DG participants were those who carried out the least level of physical activity compared to the rest of the groups and those who had the least current alcohol consumption.

Finally, data on immunometabolic biomarkers are displayed in Table 1. Significant differences were obtained between the study groups only in CRP levels. The group that had the highest levels of this inflammatory protein was the AG (*m* = 2.32; *sd* = 2.9), followed by DAG (*m* = 2.28; *sd* = 3.03), CG (*m* = 1.74; *sd* = 2.21) and DG (*m* = 1.73; *sd* = 2.57). No significant differences were observed between the groups analyzed for metabolic diseases at follow-up.

### 3.1 Metabolic and inflammatory profiles

The MIMIC model on the inflammatory score was significant and showed adequate fit indexes (χ^2^ = 36.54, *df* = 14, *p* < 0.01; RMSEA = 0.046, CFI = 0.953, TLI = 0.975, SRMR = 0.021). Parameters from the MIMIC model are included in the Supplementary Table 1, as well as correlations between the calculated inflammation score and the inflammatory biomarkers (Supplementary Figure 1). Higher inflammation scores were indicative of elevated inflammation.

**FIGURE 1:**
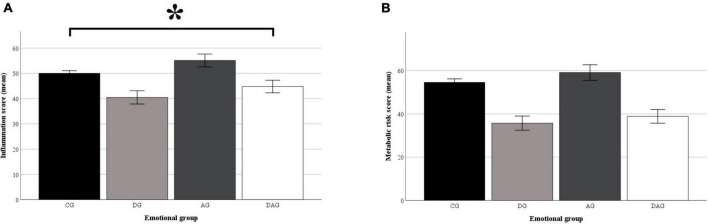
Inflammatory and metabolic risk scores according to the study groups. The inflammatory **(A)** and metabolic risk **(B)** scores were calculated using Multiple Indicators Multiple Causes (MIMIC) models. The scores were scaled on a 0–100 scale. For both scores, the higher the score the lower the inflammation/metabolic risk. Both scores were calculated controlling for lifestyle factors (i.e., Mediterranean diet adherence, physical activity and smoking), age and sex at birth. The indicators used for inflammatory score calculation were the C-reactive protein, Interleukin 6, Serum amyloid A, and Tumor necrosis factor α. Indicators used for metabolic risk calculation were waist circumference, blood glucose level, triglycerides, high-density lipoprotein level and hypertension diagnosis. CG, control group; DG, group with elevated depressive symptoms; AG, group with elevated anxiety symptoms; DAG, group with elevated depressive and anxiety symptoms. Whiskers represent the 95% confidence interval of the mean. *p < 0.05.

In terms of group comparison on the inflammatory score using linear regression, a significant relationship was found between the inflammatory score and the study groups, considering sex and age as covariates (*F*_5,749_ = 37.24, *p* < 0.001, *R*^2^*_*adj*_* = 0.19). Inflammation scores according to the study groups are displayed in the Figure 1. Specifically, the DAG group showed a significantly different loading than the CG group (*B* = 0.20, *t* = 2.03, *p* < 0.05). Sex (being a woman) (*B* = 0.20, *t* = 2.96, *p* < 0.01) and age were also significant (*B* = 0.04, *t* = 12.24, *p* < 0.001). Regression coefficients are displayed in Table 2.

**TABLE 2:** Regression loadings to explain inflammatory and metabolic risk score.

Inflammation profile model	*B*	*CI*_95_	*t*-value
**Study group (reference = control group)**			
DG	0.04	−0,16, 0.24	0.42
AG	0.17	−0.05, 0.39	1.64
DAG	0.20	0.01, 0.40	2.03*
Sex (reference = male)	0.20	0.06, 0.34	2.96**
Age	0.04	0.04, 0.04	12.24***
**Metabolic risk profile model**	***B***	**CI_95_**	***t*-value**
**Study group (reference = control group)**			
DG	0.84	−0.94, 2.62	0.92
AG	1.23	−0.63,3.09	1.30
DAG	0.91	−0.83, 2.65	1.01
Sex (reference = male)	19.26	18.06, 20.46	31.12***
Age	0.31	0.25, 0.37	11.11***

Inflammatory and metabolic risk profile model covariates: marital status, mediterranean diet adherence, physical activity level, smoking and alcohol drinking. B = Beta coefficient. CI = confidence interval at 95% of Beta coefficient. DG, depressive group; AG, anxiety group; DAG, depressive and anxiety group.

*p < 0.05;

**p < 0.01;

***p < 0.001.

The MIMIC model to estimate the metabolic risk score also showed adequate fit indexes (χ^2^ = 61.30, *df* = 21, *p* < 0.001; RMSEA = 0.050, CFI = 0.961, TLI = 0.982, SRMR = 0.062). Parameters from the metabolic model are included in the Supplementary Table 2, as well as correlations between the calculated metabolic score and the metabolic biomarkers (Supplementary Figure 1). The higher the metabolic risk score, the higher the risk of metabolic dysregulation.

Regarding the relationship between the metabolic risk profile scores and the study group and covariates, the linear regression (*F*_5,749_ = 261.2, *p* < 0.001, *R*^2^_*adj*_ = 0.63) revealed the significant relationship of the score with sex (being a woman) (being a woman) (*B* = 19.26, *t* = 31.12, *p* < 0.01) and age (*B* = 0.31, *t* = 11.11, *p* < 0.01). No significant relationships were found in terms of study groups (see Table 2 and Figure 1).

### 3.2 Metabolic diseases 10 years later

The model that showed a better fit for the disease at 10-year follow-up prediction of diabetes was the model that included all covariates (full model) (AIC = 406.05), compared to the model without covariates (AIC = 464.73) and the model that included the sociodemographic variables (AIC = 414.95). This model also showed adequate precision in the classification (AUC = 0.81).

The factors significantly related to the development of diabetes (sample in analysis, *n* = 600) were age (*OR* = 1.09, Wald’s *Z* = 5.01, *p* < 0.01) the economic status of the second (*OR* = 0.64, Wald’s *Z* = −1.99, *p* < 0.05) third (*OR* = 0.35, Wald’s *Z* = 3.25, *p* < 0.01) and fourth quartile (*OR* = 0.37, Wald’s *Z* = −3.34, *p* < 0.01), with respect to the participants of the first quartile; DG membership (*OR* = 2.08, Wald’s *Z* = 2.19, *p* < 0.05) and the metabolic risk score (*OR* = 1.05, Wald’s *Z* = 3.06, *p* < 0.01) (see Table 3).

**TABLE 3:** Logistic binary regression to predict metabolic diseases.

	OR (CI_95_)	*z*-value
**Diabetes (*n* = 600)**		
Sex (reference = male)	1.14 (0.57, 2.35)	−0.51
Age	1.09 (1.06, 1.12)	5.01**
**Household income** **(reference = 1st quartile)**		
2nd quartile	0.64 (0.3, 1.4)	−1.99*
3rd quartile	0.35 (0.17, 0.77)	−3.25**
4th quartile	0.37 (0.16, 0.86)	−3.34**
**Emotional group (reference = CG)**		
DG	2.08 (1.00, 4.22)	2.19*
AG	1.32 (0.64, 2.57)	0.97
DAG	0.6 (0.2, 1.49)	−0.22
Metabolic risk score	1.05 (1.02, 1.08)	3.06**
**Hypertension (*n* = 443)**		
Sex (reference = male)	0.06 (0.03, 0.1)	−2.21*
Age	1.04 (1.02, 1.05)	4.34**
**Household income** **(reference = 1st quartile)**		
2nd quartile	0.79 (0.49, 1.29)	−1.11
3rd quartile	0.6 (0.38, 0.97)	−1.00
4th quartile	0.83 (0.5, 1.39)	−1.53
**Emotional group (reference = CG)**		
DG	0.94 (0.55, 1.57)	1.57
AG	1.18 (0.78, 1.8)	1.19
DAG	0.98 (0.62, 1.54)	1.26
Metabolic risk score	1.26 (1.22, 1.3)	3.34**
**Hypercholesterolemia (*n* = 558)**		
Sex (reference = male)	0.93 (0.65, 1.34)	0.71
Age	1.46 (0.99, 2.16)	2.09*
**Household income** **(reference = 1st quartile)**		
2nd quartile	1.69 (1.16, 2.48)	2.51*
3rd quartile	1.74 (1.14, 2.67)	2.35*
4th quartile	1.06 (1.05, 1.07)	7.68**
**Emotional group (reference = CG)**		
DG	1.16 (0.8, 1.68)	1.01
AG	1.05 (0.74, 1.5)	1.61
DAG	1.68 (1.16, 2.43)	2.55**
Metabolic risk score	1.03 (1.02, 1.05)	2.90**

OR, odds ratio; CI, confidence interval at 95%; CG, control group; DG, depressive group; AG, anxiety group; DAG, depressive and anxiety group.

*p < 0.05.

**p < 0.01.

The full model to predict hypertension development (sample in analysis, *n* = 443) showed a better fit to data (AIC = 647.59), in comparison to the unconstrained model (AIC = 684.39) and the model that included sociodemographic variables (AIC = 658.81). The precision of the full model was considered adequate (AUC = 0.70).

The factors significantly related to the development of hypertension over the follow-up were sex (*OR* = 0.06, Wald’s *Z* = −2.21, *p* < 0.05), age (*OR* = 1.04, Wald’s *Z* = 4.34, *p* < 0.01) and metabolic risk score (*OR* = 1.26, Wald’s *Z* = 3.34, *p* < 0.01) (see Table 3).

The model without covariates (AIC = 1675.01) and the one that included the sociodemographic variables (AIC = 1536.10) showed a worse fit than the full model for hypercholesterolemia (sample in analysis, *n* = 558) (AIC = 1525.33). The full model also showed an adequate precision to predict hypercholesterolemia development (AUC = 0.73).

In Table 3, it can be seen that the variables that were significantly related to the development of hypercholesterolemia in the 10-year follow-up were age (*OR* = 1.46, Wald’s *Z* = 2.09, *p* < 0.05), second (*OR* = 1.69, Wald’s *Z* = 2.51, *p* < 0.05), third (*OR* = 1.74, Wald’s *Z* = 2.35, *p* < 0.05) and fourth financial status quartile membership (*OR* = 1.06, Wald’s *Z* = 7.68, *p* < 0.01); DAG membership (*OR* = 1.68, Wald’s *Z* = 2.55, *p* < 0.01) and metabolic risk score (*OR* = 1.03, Wald’s *Z* = 2.90, *p* < 0.01).

## 4 Discussion

This study aimed to gain insight into the relationships between subclinical profiles, featured by elevated emotional symptoms (i.e., depression and anxiety symptoms), and immunometabolic alterations. Our study involved a concurrent relationship (i.e., blood inflammatory response and metabolic biomarkers) and disease at 10-year follow-up (i.e., development of chronic metabolic diseases) approach. Individuals with symptoms of anxiety and with symptoms of anxiety and depression had higher CRP levels relative to the control group. However, no differences were observed between the groups (i.e., CG, DG, AG and DAG) in the comparison of the other inflammatory markers (i.e., IL-6, SAA and TNF-α) and metabolic markers (i.e., waist circumference, fasting glucose, triglycerides, HDL cholesterol, SBP and DBP). On the other hand, the DAG group showed higher inflammatory score and was the only group with significant differences with respect to CG. No differences were observed in the metabolic risk profiles between the different groups. At 10-year follow-up, individuals with elevated depressive symptoms had an increased risk of developing diabetes and hypercholesterolemia (in this case when comorbid anxiety systems were present) during the 10-year follow-up.

Our study provided some evidence on the influence of emotional disorders on inflammatory and metabolic function, even from subclinical statuses (i.e., statuses of elevated symptoms, regardless of other criteria to be fulfilled, such as daily interference or functional impairment). The association between inflammation and depression has been supported by studies with clinical samples (15, 25, 52–56). In fact, some emotional symptoms (i.e., anhedonia, hypervigilance, insomnia) may be conceptualized as defensive reactions against (psychological, social) pathogens, leading to increased inflammatory response (57). On the other hand, our study revealed that subclinical profiles of emotional symptoms were not associated with metabolic risk at baseline. Numerous studies have shown significant relationships between metabolic risk and emotional disorders (58, 59). The relationship between emotional disorders and metabolic risk becomes stronger in the older age, due to the effect of the progressive increase of low-grade systemic inflammation with age (60). We speculate that the statuses of full-blown disorders, qualitatively distinct from normal expression variations in both degree and kind, are needed to mobilize alterations in lipid metabolism. In the studies by van Reedt Dortland et al. (61) and Vogelzangs et al. (62), they followed this line because they found no associations with the dichotomous classification by emotional symptoms, only reporting this association in more severe patients.

Different studies support the association between major depressive disorder and the presence of MetS (12, 32, 59, 63, 64). In addition, depression has been determined as a risk factor for the development of metabolic abnormalities such as obesity and adverse patterns of lipoproteins (12). Regarding the relationship between anxiety and metabolic alterations, it has been less studied (61, 62, 65–68), not always finding positive results due to the heterogeneity of the samples and methodologies used (66–68).

Risky lifestyles, as well as the consumption of psychotropic drugs, have some relevance in the association between MetS and emotional disorders because they can alter metabolic patterns (32, 61). In addition, depression and anxiety are stress-related disorders, by which systemic cortisol action occurs along with alterations in glucocorticoid sensitivity and HPA axis action (32, 64). Derived from the deregulation of the HPA axis, the body is favored to accumulate visceral adipose tissue, an active endocrine organ that produces cytokines and inflammatory hormones (32). The greater proinflammatory response activates the release of lipids into the bloodstream, producing a reduction in HDL cholesterol together with an increase in triglycerides (64). This fact, together with the greater production of oxidative stress and dysregulation of the autonomic nervous system derived from the stressed state of the patients, interacts with the glucose homeostasis and insulin resistance, related to the factors that make up MetS (64). All these alterations produced in the organism of people with emotional disorders are those found after the association with MetS.

Patients with depression or anxiety often adopt unhealthy lifestyle habits, such as smoking (69, 70), increased intake of foods high in fat or sugar (71) or sedentary lifestyle (72, 73) that also have a determinant effect on their health status (74). In the sample of our work, we observed significant differences in physical exercise and alcohol intake. On the one hand, the DG group was the one with the highest percentage of individuals who performed little physical activity. Previous studies have shown that patients with depression perform little physical activity, with 88% of them not complying with the recommended guidelines (at least 150 min/week of moderate-intensity aerobic physical activity or 150 min/week of vigorous physical activity) (75, 76). On the other hand, it has been observed that low levels of physical activity are associated with increased risk of depression in the general population (77). In fact, people who exercise regularly show almost 45% less likelihood of having depressive symptoms, being able to be used for the prevention of this disease (78, 79).

Regarding alcohol intake, we observed that the CG subjects were more likely to consume alcohol than the other groups. In contrast, the DG group was the least likely to consume alcohol. Not consuming alcohol was associated with the diagnosis of depression in previous studies (80), and this lower alcohol consumption could be associated with the need for psychopharmacological treatment.

Statuses with comorbid elevated anxiety and depression symptoms may put individuals at higher risk of inflammatory dysregulation due to a greater impact on HPA axis function and subsequent increase of glucocorticoid resistance. In this line, Choi et al. (23) found greater HPA alterations in patients with anxious depression compared to non-anxious depression. The study by Gaspersz et al. (31) revealed an overproduction of cytokines (stimulated by increased lipopolysaccharide response) in patients with comorbid anxiety and depression. In a same vein, Shim et al. (81) observed higher levels of monocytes in patients with major depression and moderate to severe anxiety symptoms compared to the mild-anxiety symptom group. Finally, a reduced number of basophils and elevated fragmented neutrophils have been found in patients with depression who showed higher anxiety symptoms (82). Altogether, these results stress that the statuses featured by higher levels of both anxiety and depression symptoms may boost alterations in the inflammatory response at concurrent relationship.

Regarding the disease at 10-year follow-up effects of subclinical emotional statuses, our results go in line with previous studies on the relationship between the emotional disorders and metabolic disease development (83, 84). More concretely, we found that the status of elevated depression symptoms put individuals at higher risk of diabetes development over the 10-year follow-up. The status of elevated anxiety and depression symptoms was associated with hypercholesterolemia development.

The risk factors for diabetes development were age (i.e., higher age with higher risk), economic status (poorer quartiles), the status of elevated depression symptoms, and the metabolic risk at baseline. Diabetes is a serious health problem, which may contribute to the development of cardiovascular complications, stroke and subsequent early mortality (85). The total prevalence of diabetes increases significantly in relation to age, reaching figures between 10–15% in the population older than 65 years and up to 20% if we consider only those older than 80 years (86).

Mounting evidence supports the elevated comorbidity between diabetes and major depression (87–91). Our study provides further insight into this relationship, supporting a clear relationship between the status of elevated depression symptoms and diabetes. Despite this, the presence of elevated emotional symptoms of depression and anxiety together was not a risk factor. This could be due to the differences found between both groups in performing physical activity. The DAG group performs a greater amount of moderate and intense physical activity, which could be a protective factor for the development of diabetes. Physical exercise is inversely related to different risk factors for the development of diabetes (92, 93). On the one hand, it improves energy balance and reduces adiposity and, in addition to this, it improves insulin sensitivity and glucose homeostasis, which helps improve the metabolic profile of people who do it and reduce the risk of diabetes (92).

On the other hand, the risk factors for the disease at 10-year follow-up development of hypertension were sex, age and metabolic risk. The statuses of elevated emotional symptoms were not associated with hypertension development. Despite some studies have provided some evidence on the relationship between emotional disorders (mainly depression) and hypertension (94–96), other results have shown opposite findings (97–99), being important to consider confounding factors such as lifestyle or metabolic status of the participants (100). Moreover, some cardiovascular mediating paths (i.e., kynurenine path) may be more independent of emotional factors (28).

Finally, the status of elevated anxiety and depression symptoms was proven to be a risk factor of hypercholesterolemia development. Additional risk factors of hypercholesterolemia development were age, economic status (poorer quartiles), and the metabolic risk at baseline.

Depression has been related with altered lipid metabolism (101), even from a first clinical episode (102). Despite the fact that many studies support this fact (35, 103, 104), the results are contradictory with other studies that have found an inverse relationship between cholesterol and depression (105, 106). These discrepancies may be explained by methodological issues (i.e., different sample selection criteria and assessment protocols).

The longitudinal study by van Reedt Dortland et al. (35) stressed that patients with severe anxiety and depression symptoms were at higher risk of presenting dyslipidemia on a 2-year follow-up. Our results extend the conclusions from the study by van Reedt Dortland et al. (35), by including a longer follow-up and individuals with subclinical statuses of elevated symptoms. We speculate that the individuals with statuses of elevated emotional may show an overproduction of HPA agents and higher glucocorticoid resistance. HPA dysregulation may lead to increased levels of circulating free fatty acids, with subsequent low-density lipoprotein secretion and alterations in lipid metabolism (101).

Our study presents some relevant strengths to be mentioned. Compared to previous studies, our study has a large sample of community people. Moreover, our analytical strategy based on robust protocols (e.g., MIMIC models) controlling for relevant covariates, such as, lifestyle factors and health status. Finally, our study focuses on profiles on symptoms, providing new insight into the development of preventive strategies to prevent from full-blown condition development.

Our study presents some shortcomings to be mentioned. The intake of oral hypoglycemic, antihypertensive or lipid-lowering drugs was not taken into account in the baseline evaluation. Data on anti-inflammatory drugs and psychopharmacological medication were also not collected in this study. In this regard, we adopted a symptom-based approach, highly appropriate on a community basis. However, this study should be seen as a wide picture of how subclinical statuses of emotional disorder may be linked with immunometabolic dysregulation and metabolic diseases. On the other hand, it should be noted that only a baseline assessment of the participants’ mental health was carried out. Anxiety and depressive symptoms were not followed up, so the trajectories of the participants’ symptoms could not be known. Longitudinal studies are needed to explore how different trajectories of anxiety and depression symptoms influence the subsequent development of metabolic diseases.

## 5 Conclusion

Depression and anxiety are two of the most prevalent (1) and disabling (5, 6) mental disorders that carry high socioeconomic costs. Therefore, finding the causes of both disorders to reduce or eliminate symptoms is essential in mental health research. With our study, we have been able to demonstrate that both pathologies have concurrent relationship and disease at 10-year follow-up consequences on the health of individuals. We have determined that subjects with comorbid subclinical symptoms of depression and anxiety have concurrent relationship immune system consequences. In addition, these patients have a higher risk of long-term hypercholesterolemia and patients with depression have a higher risk of diabetes. The results therefore suggest the need to follow these patients and propose early healthy lifestyle interventions that can offset this risk by reducing their metabolic risk and thereby reducing the risk of morbidity.

## Data availability statement

The raw data supporting the conclusions of this article will be made available by the authors, without undue reservation.

## Ethics statement

The ATTICA study was approved by the Institutional Ethics Committee of Athens Medical School (#017/1.5.2001). The studies were conducted in accordance with the local legislation and institutional requirements. The participants provided their written informed consent to participate in this study. Written informed consent was not obtained from the individual(s) for the publication of any potentially identifiable images or data included in this article because protocols in the current study did not need a specific approval from an Ethics Committee as secondary data analysis was performed.

## Author contributions

DP and CP designed the study and wrote the protocol. YS-C and AT-L performed the statistical analyses. YS-C managed the literature searches and wrote the first draft of the manuscript, which was supervised by AT-L, PL-G, and JA-M. EG and CV participated in the recruitment, the collection of information, and the creation of databases. All authors contributed to the interpretation and discussion of the results and have approved the final manuscript.
