# Supplementary material for: Effects of elevated emotional symptoms on metabolic disease development: a 10-year follow-up study
Source: Front Psychiatry. 2023 Dec 4;14:1148643. doi: 10.3389/fpsyt.2023.1148643 (PMC10725934; doi:10.3389/fpsyt.2023.1148643)
Supplement: Supplementary file 1 [file Data_Sheet_1.pdf]

Supplementary material

Table S1. Parameters derived from the Multiple Indicators Multiple Causes Model for the inflammation score.

	Estimate	*SE*	*Z*	*p*	Standardized estimate
Loading (*B*)					
CRP*	1				0.805
Il-6	0.197	0.013	15.449	0	0.928
SAA	0.262	0.034	7.721	0	0.352
TNF-α	0.235	0.022	10.816	0	0.56
Regressor					
MedDietScore	-0.035	0.006	-5.97	0	-0.264
Physical Activity level	-0.195	0.037	-5.245	0	-0.16
smoking	0.162	0.081	2.003	0.045	0.078
alcohol	-0.025	0.129	-0.196	0.845	-0.008
Intercept					
CRP*	0				0
Il-6	0.339	0.026	12.875	0	1.569
SAA	1.014	0.144	7.062	0	1.332
TNF-α	2.209	0.072	30.613	0	5.15
Inflammatory score	0.683	0.239	2.86	0.004	0.668
Variance					
CRP	0.568	0.066	8.616	0	0.353
Il-6	0.006	0.002	2.785	0.005	0.138
SAA	0.508	0.033	15.615	0	0.876
TNF-α	0.126	0.016	7.755	0	0.687
Inflammatory score	0.934	0.083	11.317	0	0.896

Note. The inflammatory indicators were transformed into loglinear scale.
SE = Standard error. CRP = C-reactive protein. iL-6 = Interleukin 6. SAA = Serum amyloid A. TNF-α = Tumour necrosis factor α.
* Fixed due to model identification requirements.

Table S2. Parameters derived from the Multiple Indicators Multiple Causes Model for the metabolic risk score.

	Estimate	*SE*	*Z*	*p*	Standardized estimate
Loading (*B*)					
Waist circumference*	1				0.725
Glucose level	0.004	0.001	4.13	0	0.276
Triglyceride	0.028	0.003	8.205	0	0.653
HDL	-0.011	0.001	-7.309	0	-0.495
Hypertension	0.055	0.007	7.677	0	0.625
Regressor					
Mediterranean diet[1]	-0.594	0.075	-7.904	0	-0.387
Physical activity	-1.797	0.552	-3.254	0.001	-0.127
Smoking (ref.= no)	3.905	0.981	3.98	0	0.162
Alcohol (ref.= no)	1.283	1.647	0.779	0.436	0.034
Intercept					
Waist circumference*	0				0
Glucose level	4.128	0.104	39.575	0	23.614
Triglyceride	1.744	0.367	4.757	0	3.388
HDL	4.923	0.155	31.7	0	19.104
Hypertension*	0				0
Metabolic risk score	105.263	2.991	35.189	0	8.902
Threshold					
Hypertension*	5.186	0.779	6.654	0	4.969
Variance					
Waist circumference*	125.984	23.117	5.45	0	0.474
Glucose level	0.028	0.004	7.375	0	0.924
Triglyceride	0.152	0.013	11.63	0	0.574
HDL	0.05	0.004	14.193	0	0.755
Hypertension*	0.664				0.61
Metabolic risk score	110.433	16.174	6.828	0	0.79

Note. Waist circumference, blood glucose level, triglycerides and HDL were transformed into loglinear scale. Hypertension was operatized as a binary variable (levels = yes/no) *SE* = Standard error. HDL = High-density lipoprotein.

[1] Measured by the Mediterranean Diet Adherence Screener test.

* Fixed due to model identification requirements.

Figure S1. Correlations between the inflammation and metabolic risk scores and related biomarkers.

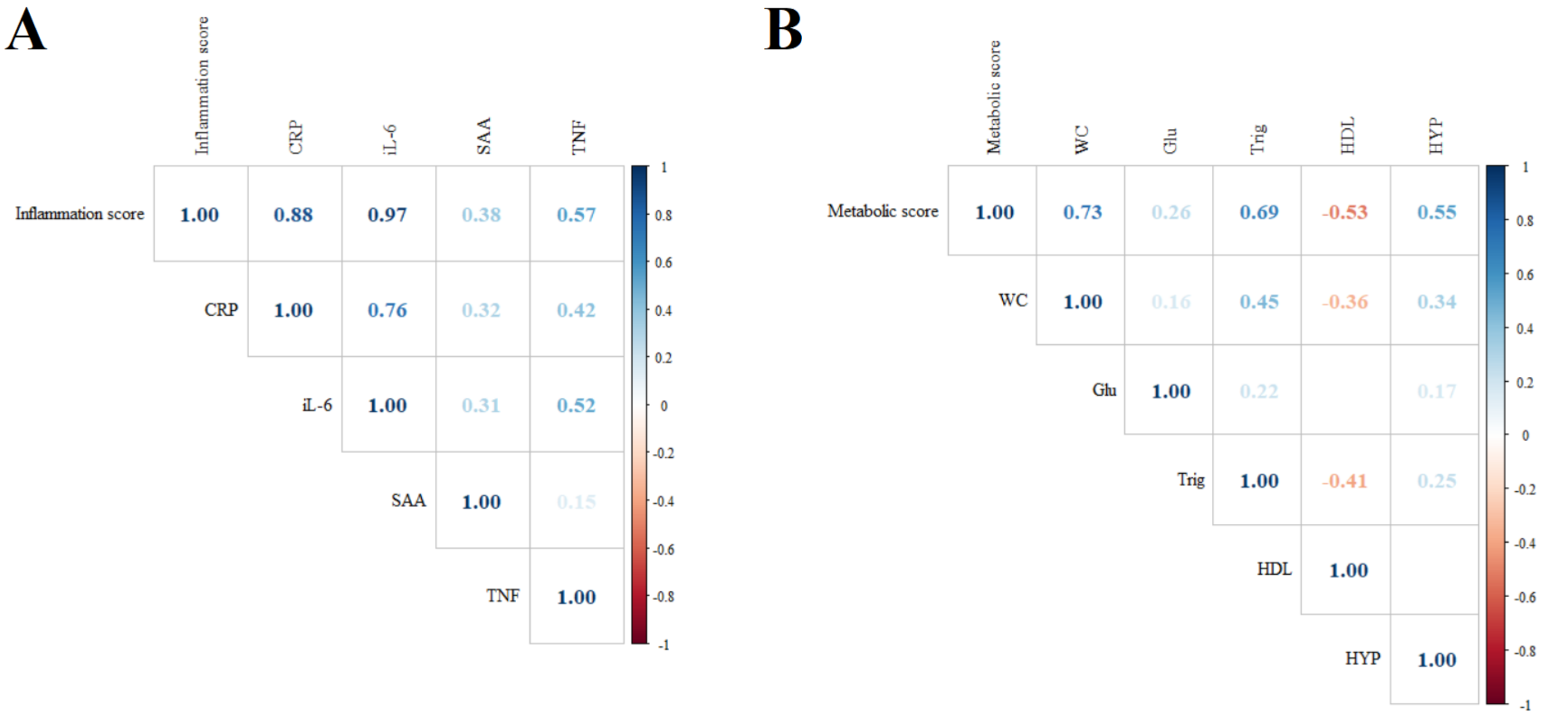

Note. Pearson's *r* correlations between the inflammatory indicators (in loglinear scale) and the inflammation scores are displayed in box A. Pearson's *r* correlations between the metabolic indicators (three in loglinear scale: Glu, Trig and HDL levels) and the metabolic risk score are displayed in box B. The point-biserial *r* estimate was calculated to study the relations between the hypertension diagnosis indicator with the other indicators and the metabolic risk score. All the displayed correlations were significant ($p < .05$).

CRP = C-reactive protein. iL-6 = Interleukin 6. SAA = Serum amyloid A. TNF = Tumour necrosis factor α. WC = Waist circumference. Glu= Blood glucose. Trig = Triglycerides. HDL = High-density lipoprotein. HYP = Hypertension diagnosis.
